# Reduction of miR-744 delivered by NSCLC cell-derived extracellular vesicles upregulates SUV39H1 to promote NSCLC progression via activation of the Smad9/BMP9 axis

**DOI:** 10.1186/s12967-020-02654-9

**Published:** 2021-01-20

**Authors:** Liming Gao, Qi Tian, Tong Wu, Shanshan Shi, Xiaobo Yin, Lijie Liu, Lei Zheng, Ping Wang, Yaling Tian, Shufeng Xu

**Affiliations:** 1 Department of Oncology, The First Hospital of Qinhuangdao, Qinhuangdao, 066000 P.R. China; 2 Department of Respiratory, The First Hospital of Qinhuangdao, No. 258, Wenhua Road, Haigang District, Qinhuangdao, 066000 Hebei P.R. China; 3 Hebei Medical University, Shijiazhuang, 050000 P.R. China; 4 Chengde Medical College, Chengde, 067000 P.R. China; 5 Department of Respiratory, Chinese PLA General Hospital, Beijing, 100853 P.R. China

**Keywords:** NSCLC, miR-744, SUV39H1, Smad9, BMP4

## Abstract

**Background:**

Non-small cell lung cancer (NSCLC) is a common type of lung cancer. Extracellular vehicles (EVs) are nano-sized particles containing proteins, lipids, and miRNAs secreted by various cells, which play important roles in the development of lung cancer by regulating a wide range of signaling pathways. This study focused on elucidating a potential mechanism by which EVs promote the development of NSCLC.

**Methods:**

Expression levels of miR-744, SUV39H1, Smad9, and BMP4 in clinical tissue samples of NSCLC patients and cell lines were quantified by RT-qPCR and/or western blot analysis. The interaction between SUV39H1 and miR-744 was identified by dual-luciferase reporter assay. miR-744, SUV39H1, and BMP4 expression levels were modulated in A549 and H460 cells, in order to evaluate their effects on cell proliferation, colony formation and cell cycle. A NSCLC xenograft mouse model was used to verify the in vitro findings. NSCLC cell-derived EVs and normal bronchial epithelial cell-derived EVs were isolated and their roles in NSCLC development were evaluated in vivo and in vitro.

**Results:**

miR-744 expression was lower in cancer cell-derived derived EVs than in normal lung epithelial cell-derived EVs. Reduced miR-744 expression in EVs upregulated SUV39H1 in NSCLC cells and further increased BMP4 levels to promote NSCLC development. BMP4 was upregulated in NSCLC cells upon suppression of Smad9 mediated by SUV39H1. Reduced miR-744 expression transferred from cancer cell-derived EVs into NSCLC cells enhanced cancer development.

**Conclusion:**

Overall, our findings unveiled a mechanism whereby miR-744 delivered by NSCLC-derived EVs upregulated SUV39H1, activating the Smad9/BMP9 axis and thus promoted cancer development.

## Background

Lung cancer is the most common cause of cancer-related deaths in the world [1]. Non-small cell lung cancer (NSCLC) accounts for approximately 85% of all lung cancers, and has a 5-year survival rate less than 15% [2]. Therefore, it remains critical to understand the pathogenesis of NSCLC and to discover novel therapeutic targets that may be leveraged for treatment.

Extracellular vesicles (EVs) comprise of nano-sized particles secreted by different types of cells into the microenvironment. Carrying a variety of bioactive molecules such as proteins, lipids, and nucleic acids including microRNAs (miRNAs), EVs are actively involved in both physiological and pathological processes [3–5]. For instance, cancer cell-derived EVs have been shown to regulate the metastasis, angiogenesis, proliferation, and chemosensitivity of NSCLC [6–9]. Though accumulating evidence points to the importance of EVs in NSCLC development and progression, the underlying molecular mechanisms remain elusive.

miRNAs and EVs are closely involved in cancer pathogenesis. miRNAs can be packaged in EVs and exert regulatory functions on tumorigenesis. For instance, breast cancer cells secrete EVs, which can deliver miRNAs to normal mammary epithelial cells and induce neoplastic transformation [10]. Further evidence has demonstrated that EV-delivered miR-222-3p promotes NSCLC by increasing cancer cell proliferation and migration [11]. EV-derived miR-96 has also been shown to promote lung cancer cell proliferation and migration by targeting LIM-domain-only protein 7 (LMO7). Interestingly, targeting miR-96 resulted in reduced cell proliferation and migration in lung cancer cells [12]. Together, these findings highlight the importance of EV-derived miRNAs in cancer development. In another instance, miR-744, which has been shown to inhibit NSCLC proliferation [13], has also been shown to be downregulated in NSCLC cell-derived EVs and has been considered an early diagnostic biomarker for NSCLC [14]. However, the mechanisms underpinning the involvement of downregulated miR-744 in cancer cell-derived EVs in the development of NSCLC has not been studied.

Suppressor of variegation 3-9 homolog 1 (SUV39H1), which is known as a histone methyltransferase, has been implicated in the development of NSCLC. A recent study has shown that silencing SUV39H1 led to elevated NSCLC cell proliferation, migration, and survival [15]. On the other hand, reduced expression of SUV39H1 is associated with upregulation of Smad9 [16], a negative regulator of bone morphogenetic protein 4 (BMP4) [17]. Notably, recent evidence has suggested that BMP4 promotes the tumorigenesis of NSCLC [18]. Based on these reports, it appears reasonable to speculate that SUV39H1 is associated with NSCLC tumorigenesis in a Smad9/BMP4 dependent manner.

In the current study, we have confirmed that miR-744 expression in NSCLC cell derived EVs was reduced compared with that in normal non-tumor cell derived EVs. Target gene screening and validation demonstrated that the histone methyltransferase SUV39H1 is the target gene of miR-744. Moreover, our data showed that reduced miR-744 in cancer cell-derived EVs contributes to upregulation of SUV39H1. Furthermore, our findings showed that upregulated SUV39H1 positively regulates the expression of BMP4 to promote NSCLC tumorigenesis by suppressing Smad9 in NSCLC cells. Thus, our study has connected the gap between downregulated miR-744 in tumor cell-derived EVs and upregulated SUV39H1 promoted NSCLC development. This study will improve our understanding of the pathogenesis of NSCLC and will also contribute to the identification of novel therapeutic target toward NSCLC.

## Materials and methods

### Ethics statement

The study protocol was ratified by the Ethics Committee of the First Hospital of Qinhuangdao and conformed to the *Declaration of Helsinki*. All participants provided signed written informed consent documentation. Experiments involving animals were implemented under ratification from the Animal Ethics Committee of the First Hospital of Qinhuangdao strictly following the Guide for the Care and Use of Laboratory Animals published by the US National Institutes of Health. Best efforts were made to minimize the numbers and suffering of the included animals.

### Cell culture

Human NSCLC cell lines (H1299, A549, PC-9, H358, H522, and H460) and normal bronchial epithelial cells (BEAS-2B) were obtained from Shanghai Institute of Biochemistry and Cell Biology (Shanghai, China). NSCLC cell culture was implemented in Dulbecco’s modified Eagle’s medium (Gibco, Grand Island, NY, USA) encompassing 10% fetal bovine serum (FBS), 100 U/mL penicillin and 100 μg/mL streptomycin (Gibco). BEAS-2B cell incubation was conducted in LHC-9 complete medium (Gibco). All cells were maintained at 37˚C under 5% CO_2_.

### EV isolation

Extraction of EVs was conducted based on a previously described method [19]. Briefly, NSCLC cells (H1299 and H522 cells) and BEAS-2B cells were cultured in medium without serum for 72 h and the supernatants were attained following 10-min centrifugation at 3000 g and 4 ℃. Thereafter, large EVs were removed by 30-min centrifugation of the supernatants at 10,000 g and 4 ℃. Then, microvesicles and apoptotic bodies were discarded by passing the supernatants through a 0.22 μm filter (Millipore, Burlington, MA, USA). The 2-h ultracentrifugation was performed subsequently (100,000 g, 4 ℃) (Optima L-80XP, Beckman Coulter, Brea, CA, USA) and the supernatants were carefully discarded. The obtained pellets were suspended in ice-cold phosphate-buffered saline (PBS) and ultracentrifugation was repeated. After centrifugation, PBS was removed and the EVs were suspended in 200 μL ice-cold PBS.

### EV characterization

The size distribution of EVs was identified by Nanoparticle tracking analysis (NTA). Subsequent to dilution and mixing in 1 mL PBS, EVs were loaded into Nanosight NS300 instrument (Malvern, UK). The particle size was assessed based on Brownian motion and the diffusion coefficient as reported [20]. Next, the morphology of EVs was observed using transmission electron microscopy (TEM). Briefly, 5-min EV (10 μL) incubation was conducted on formvar carbon-coated 200 mesh copper electron microscopy grids at ambient temperature. Thereafter, EVs were stained with 1% uranyl acetate for 1 min at ambient temperature, and then semi-dried at ambient temperature prior to TEM (Hitachi H-7650, Hitachi, Tokyo, Japan) observation. EV specific markers CD63 (ab216130, rabbit antibody, 1:2,000, Abcam, Cambridge, UK), Tumor Susceptibility Gene 101 (TSG101; ab125011, rabbit antibody, 1:10,000, Abcam), CD81 (ab109201, rabbit antibody, 1:10,000, Abcam) and Calnexin (ab92573, rabbit antibody, 1:100,000, Abcam) were finally detected through western blot analysis in order to identify the characteristics of the isolated EVs.

### Immunofluorescence assay

Isolated EVs were tagged with PKH67 green fluorescence dye (PKH67, Sigma-Aldrich, St Louis, MO, USA) in the light of the manufacturer’s directions. Cells were seeded into 8-well chamber slides (Thermo Fisher Scientific, Rockford, IL, USA) at a concentration of 8000 cells/well, then added with 5 μL PKH67-labeled EVs, and incubated for 4 h for internalization. Next, the slides were fixed with 4% paraformaldehyde (Beijing Leagene Biotech. Co., Ltd., Beijing, China) for 15 min. EV specific marker protein CD63 (ab217345, 1:100, Abcam) was supplemented for overnight incubation of slides at 4 ℃. The next day, the red fluorescence-labeled secondary antibody was supplemented for 1-h incubation of slides in the dark at ambient temperature. Subsequent to nuclei staining with 4′,6-diamidino-2-phenylindole, the slides were observed, and images were obtained using a Zeiss LSM 780 (Zeiss, Germany) confocal microscope.

### Clinical samples

NSCLC and normal adjacent tissues were harvested from 20 patients receiving digestive tract surgery at the First Hospital of Qinhuangdao between 2015 and 2019. All the included patients had not undergo any anti-tumor therapies prior to diagnosis.

### Cell transfection and treatment

Lipofectamine 3000 reagents (Invitrogen, Carlsbad, CA, USA) were used to transfect NSCLC cells (A549, H1299, and H522 cells) and BEAS-2B cells as per the manufacturer’s directions. A549 cells were transfected with miR-744 mimic or inhibitor (Ambion, Carlsbad, CA, USA), overexpression (OE) plasmids (OE-SUV39H1, OE-Smad9) (GeneChem, Shanghai, China), or small interfering RNAs (siRNAs) (si-SUV39H1, si-Smad9, or si-BMP4) (RiboBio, Guangdong, China), respectively. G418 (Solarbio, Beijing, China) was used to select A549 cells for stable Smad9 expression. Based on the treatment and/or transfected constructs, A549 cells were arranged into 8 batches of groups: (1) BEAS-2B-EVs, H1299-EVs, H522-EVs; (2) mimic-negative control (NC), miR-744 mimic; (3) H1299-EVs-NC, H522-EVs-NC, H1299-EVs-miR-744, H522-EVs-miR-744; (4) mimic-NC, miR-744 mimic, inhibitor-NC, miR-744 inhibitor; (5) BEAS-2B-EVs + si-NC, H1299-EVs + si-NC, si-SUB39H1 + BEAS-2B-EVs, si-SUV39H1 + H1299-EVs; (6) si-NC, si-SUV39H1, si-Smad9, si-SUV39H1 + si-Smad9; (7) BEAS-2B-EVs + OE-NC, BEAS-2B-EVs + OE-Smad9, H1299-EVs + OE-NC, H1299-EVs + OE-Smad9; (8) BEAS-2B-EVs + si-NC, H1299-EVs + si-NC, BEAS-2B-EVs + si-BMP4, H1299-EVs + si-BMP4.

### Cell counting kit-8 (CCK-8) assay for cell proliferation [21]

Cells were seeded into 96-well plates at 5,000 cells/well. Thereafter, 20 μg/mL EVs isolated from the BEAS-2B, H1299 and H522 cells were incubated with A549 and H460 cells, and at 0, 24, 48 and 72 h, 10 μL CCK-8 solution (Dojindo, Kyushu Island, Japan) and 100 μL fresh medium were supplemented into each well. After 1 h of incubation at 37 ℃, the optical density (OD) value at 450 nm was measured using a microplate reader (Bio-Rad 680, Bio-Rad). The average OD values from each group were used to reflect the cell proliferation.

### Flow cytometry-based cell cycle analysis [20]

Following 48-h EV treatment, cells were attained. Then Annexin V/propidium iodide (PI) assay kit (BD Biosciences, Franklin Lakes, NJ, USA) was utilized in the assay. In short, 0.5 μg/mL fluorescein isothiocyanate (FITC)-Annexin V and 5 μg/mL PI were applied for double staining at ambient temperature for 30 min. A flow cytometer (FACScan, BD Biosciences) equipped with Cell Quest 3.0 software was employed to analyze and calculate the percentage of cells at different stages. For cell cycle analysis, PI staining of the treated cells was implemented using CycleTEST TM PLUS DNA kit (BD Biosciences), and according to the provided instructions, the percentage of the cells at the G0/G1, G2/M and S phases was calculated and compared.

### Flow cytometry-based cell apoptosis analysis

After treatment, the cells were detached with 0.25% trypsin without EDTA (YB15050057, Shanghai Yubo Biotechnology Co., Ltd., Shanghai, China), positioned in flow tubes and centrifuged, after which the supernatant was discarded. Subsequent to centrifugation, the supernatant was removed. The Annexin-V-FITC cell apoptosis detection kit (K201-100, BioVision Milpitas, CA, USA) was used, and Annexin-V-FITC, PI and 4-(2-hydroxyethyl)-1-piperazineëthanesulfonic acid (HEPES) buffer solution were prepared into Annexin-V-FITC/PI staining solution at the ratio of 1:2:50, based on the manufacturer’s instructions. Every 100 μL staining solution was adopted to re-suspend 1 × 10^6^ cells, which were mixed by shaking. Subsequent to 15-min incubation at ambient temperature, cells were supplemented with 1 mL HEPES (PB180325, Procell Life Science & Technology Co., Ltd., Wuhan, Hubei, China) and mixed by shaking. Subsequent to initiation by excitation at 488 nm, fluorescence measurement was implemented with emission filters at 525 nm (FITC) and 620 nm (PI) to determine cell apoptosis.

### Scratch test

The 24-h cell incubation was conducted in a 6-well plate at 2.5 × 10^4^ cells/cm^2^. Thereafter, the medium was removed, and a 10 μL sterile disposable straw was used to scratch. After 2 washes with PBS, the cells were subjected to additional culture in the FBS-free medium, and the images of each well were captured at 0 and 48 h post scratch under an inverted microscope, with 3 replicates set in each group. Image J software was adopted to determine the width of each scratch, and the cell migration ability was evaluated by comparing the scratch width of each group. The ratio of scratch healing rate = (scratch width at 0 h–scratch width at 48 h)/scratch width at 0 h × 100%.

### Colony formation assay

A549 and H460 cells under different treatments were cultured in 6-well plates at 1000 cells/well for 2 weeks. Cells underwent 20-min 100% absolute methanol fixing. After fixation, 10-min cell staining was implemented with 0.1% crystal violet (G1064, Solarbio). The number of colonies with more than 50 cells was counted under a microscope (Leica, Germany). Counting was repeated three times for each group.

### Bioinformatics analysis

Expression of miR-744 in lung cancer and normal samples was compared using the miRNA Pathway website (http://bioinfo.life.hust.edu.cn/miR_path/). The target genes of miR-744 were then predicted using miRSearch (https://www.exiqon.com/miRSearch), TargetScan (http://www.targetscan.org/vert_72/), and miRWalk databases (http://mirwalk.umm.uni-heidelberg.de/). The predicted target genes were then visualized using a Venn diagram drawn using online tools (http://bioinformatics.psb.ugent.be/webtools/Venn/). Differentially expressed regulatory factors related to miR-744 in lung cancer were analyzed using the GEPIA database (http://gepia.cancer-pku.cn/). Amongst these, target genes with opposite expression trend were considered as the competing endogenous RNA (ceRNA) of miR-744.

### Human NSCLC Xenografts in nude mice

Establishment of a xenograft model was performed as previously described [22]. A total of 56 male BALB/c nude mice (aged 6–8 weeks old; Shanghai SLAC Laboratory Animal Co., Ltd., Shanghai, China) were selected for this study. Each mouse received a subcutaneous injection of 150 μL PBS encompassing 5 × 10^6^ NSCLC cells into the unilateral dorsal flank area (n = 8/group). Seven days after transplantation, EVs (10 μg) were injected into the tumor every other day. Measurement of the subcutaneous tumors was performed every three days. Tumor volume was calculated: V = LW^2^/2, where L and W represent tumor length and width (in mm), respectively. The mice were euthanized after 4 weeks whereupon the tumors were harvested and weighed. The harvested tumor tissues were subjected to histologic analysis and immunohistochemical staining.

### Histological analysis

Tumor tissues from the xenografts were fixed using formalin, paraffin-embedded, and then sliced. The slices were subjected to hematoxylin–eosin (HE) staining for histological evaluation and immunohistochemistry (IHC) staining for Ki-67 detection. HE staining was conducted using HE Staining Kit (Beyotime, Shanghai, China) as per the manufacturer’s manuals. For Ki-67 detection, the tissue sections were stained in the light of the protocols of streptavidin biotin peroxidase complex-POD IHC kit with primary rabbit antibody against Ki-67 (A2094, abclonal, Woburn, MA, USA, 1:100).

### Dual-luciferase reporter assay

Wild type (WT) or mutant type (MUT) 3′ untranslated region (3′UTR) of SUV39H1 encompassing the predicted target sequence of miR-744 were engineered into the pGL3 promoter vector (Genscript, Nanjing, China) and a dual-luciferase reporter assay was performed as previously reported [23]. Briefly, HEK293T cells (American Type Culture Collection, Manassas, VA, USA) were seeded into 24-well plates at 5 × 10^5^ cells/well one day prior to transfection. The following day, 0.12 μg luciferase reporter vectors were co-transfected with miR-744 mimic or mimic-NC into HEK293T cells using Lipofectamine 3000 reagents. Forty-eight hours post-transfection, luciferase activity determination was implemented on a luminometer as per the provided protocol. Luciferase activity was calculated: luciferase activity = luciferase activity of firefly/luciferase activity of Renilla.

### Reverse transcription quantitative polymerase chain reaction (RT-qPCR)

TRIzol reagents (Invitrogen) were applied for total RNA isolation from NSCLC cells. For mRNA quantification, cDNA was generated using Revert Aid first-strand cDNA synthesis kit (Fermentas) from 1 μg total RNA. RT-qPCR was implemented in an ABI PRISM 7900 System using SYBR Premix ExTaq™ II (Takara, Kyoto, Japan) with glyceraldehyde-3-phosphate dehydrogenase (GAPDH) as a normalizer. miRNAs from EVs were extracted using SeraMir EVssome RNA Purification Kit (System Biosciences, San Francisco, CA, USA) underwent reverse transcription to generate cDNA using TaqMan microRNA assay kit (Applied Biosystems, Carlsbad, CA, USA). The samples were analyzed using FastStart Universal SYBR Green Master Mix (Roche, Indianapolis, IN, USA) with miRNA-specific forward primer (Sangon, Shanghai, China) and universal reverse primer provided by the TaqMan microRNA assay kit. U6 was adopted as a normalizer. All primers used in this study are listed in Table 1.

**Table 1:** Primer sequences for RT-qPCR reaction

Targets	Forward primer sequence (5′–3′)	Reverse primer sequence (5′–3′)
miR-744	AATGCGGGGCTAGGGCTA	GTGCAGGGTCCGAGGT
U6	CGGGTTTGTTTTGCATTTCT	AGTCCCAGCATGAACAGCTT
SUV39H1	TGCGTATCCTCAAGCAGTTCC	CCGTCCAGGTCCACCTCATTC
GAPDH	CGGATTTGGTCGTATTG	GAAGATGGTGATGGGATT
Smad9	CTTTCCAGCAGCCTCCGTGC	GGGGTGGTGTGTCAACTGAGTG
BMP4	TAGTCCCAAGCATCACCC	TCTCAGCGGCATCCAC

### Western blot analysis

Subsequent to isolation from tissues or cells using lysis buffer encompassing protease inhibitor cocktail, the total protein was then separated by sodium dodecyl sulfate polyacrylamide gel electrophoresis and electroblotted onto polyvinylidene fluoride membranes. Subsequent to 1-h membrane blocking with 5% skimmed milk powder at ambient temperature, overnight membrane incubation was implemented with primary rabbit antibodies against SUV39H1 (A3277, abclonal, 1: 2,000), Smad9 (ab96698, 1: 1,000, Abcam), BMP4 (A11315, abclonal, 1: 2,000), phosphorylated (p)-Smad9 (#13820, 1: 1,000, Cell Signaling Technologies, Beverly, MA, USA), and GAPDH (AC033, abclonal, 1: 50,000) at 4 ℃. The next day, the membranes were re-probed with horseradish peroxidase-conjugated secondary antibodies against rabbit immunoglobulin G (IgG) (AS014, abclonal, 1:10,000) or mouse IgG (A3950, abclonal, 1:10,000) at ambient temperature for 1 h. Subsequent to development using enhanced chemiluminescence reagents (Thermo Fisher Scientific), images were obtained using a ChemiDox XRS Plus luminescent image analyzer (Bio-Rad Laboratories, Hercules, CA, USA). The obtained images were then analyzed using Image-Pro Plus 6.0 software (three repeated experiments). GAPDH was used as a loading control.

### Statistical analysis

Data analysis was conducted using SPSS 21.0 software (IBM Corp. Armonk, NY, USA). Measurement data were summarized as mean ± standard deviation. Data comparisons between two groups were made using unpaired *t-*test, while data from multiple groups was compared using one-way analysis of variance (ANOVA) with Tukey’s post hoc tests. Comparisons of data obtained at multiple time points were conducted using Bonferroni-corrected repeated measures ANOVA. A value of *p* < 0.05 was considered statistically significant.

## Results

### Cancer cell-derived EVs promoted the proliferation of NSCLC in vivo and in vitro

**Fig. 1:**
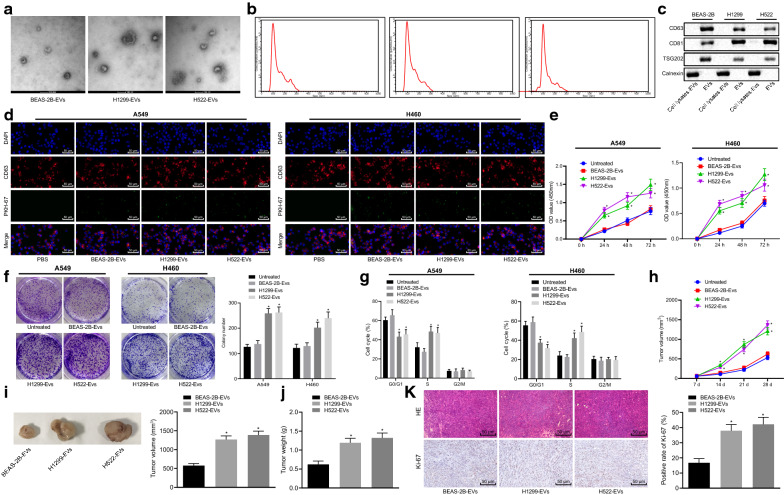
Cancer cell-derived EVs promote NSCLC cell proliferation in vitro*.* **a** Morphology of the isolated EVs from the BEAS-2B, H1299, and H522 cells observed using TEM (scale bar: 100 nm). **b** EV size distribution analyzed by NAT. **c** EV surface markers evaluated by western blot analysis. **d** Representative images showing the EV internalization. After the cells were fixed, they were stained with CD63 antibody, followed by EV tracking (× 200). **e** Viability of untreated A549 and H460 cells and A549 and H460 cells treated with EVs isolated from BEAS-2B, H1299 and H522 cells measured by CCK-8 assay. **f** Colony formation of untreated A549 and H460 cells and A549 and H460 cells treated with EVs isolated from BEAS-2B, H1299 and H522 cells measured by colony formation assay. **g** Cell cycle distribution in untreated A549 and H460 cells and A549 and H460 cells treated with EVs isolated from BEAS-2B, H1299 and H522 cells measured by flow cytometry. **h** Tumor growth curve in the xenograft model mice injected with untreated A549 cells and A549 cells treated with EVs isolated from BEAS-2B, H1299 and H522 cells. **i** Representative images showing xenografts in nude mice injected with A549 cells treated with EVs isolated from BEAS-2B, H1299 and H522 cells. **j** Tumor weight of mice injected with A549 cells treated with EVs isolated from BEAS-2B, H1299 and H522 cells. **k** HE staining (the upper panel) showing tumor development and IHC staining (the lower panel) showing the expression of Ki-67 (× 200). Mice: n = 8/group, * *p* < 0.05 vs. untreated A549 and H460 cells, EVs isolated from BEAS-2B cells, or mice treated with untreated A549 cells. Data are shown as mean ± standard deviation. Data from multiple groups were assessed using one-way ANOVA with Tukey's tests. Data at difference time points were assessed by Bonferroni-corrected repeated measures ANOVA. All experiments were repeated three times independently

EVs were isolated from the medium of normal bronchial epithelial cells (BEAS-2B) and NSCLC cells (H1299 and H522) and characterized. TEM was used to observe the morphology of the isolated EVs, which revealed that the EVs displayed round-shaped structures (Fig. 1a). The diameter of the isolated EVs analyzed using NTA was found to range from 40 to 120 nm (Fig. 1b). Further, western blot analysis showed that EVs expressed high levels of CD63, CD81, and TSG101, which are typical surface markers of EVs, but no Calnexin, which is a marker of endoplasmic reticulum (Fig. 1c). These results indicated the successful isolation of EVs. To confirm that these EVs could be delivered into host cells, we tracked the PKH67 labeled EVs (green fluorescence) by incubating them with A549 and H460 cells. After 4 h of incubation, green fluorescence could be observed in the cells (Fig. 1d), indicating that EVs had entered the host cells. We hypothesized that cancer cell-derived EVs could promote the proliferation of NSCLC cells and thus, we performed CCK-8 proliferation and colony formation assays. The viability of untreated A549 and H460 cells was comparable to that of A549 and H460 cells treated with normal bronchial epithelial cell-derived EVs (BEAS-2B-EVs). Compared with BEAS-2B-EVs, cancer cell-derived EVs (H1299-EVs or H522-EVs) increased NSCLC cell (A549 and H460) viability (Fig. 1e). In agreement, the results of the colony formation assay also showed that no obvious difference was observed in colony formation between untreated A549 and H460 cells and BEAS-2B-EV-treated A549 and H460 cells. Upon treatment with H1299-EVs or H522-EVs, NSCLC cells (A549 and H460) formed more colonies than upon treatment with BEAS-2B-EVs (Fig. 1f). As reflected by flow cytometry, the cell cycle distribution of untreated A549 and H460 cells was almost equivalent to that of BEAS-2B-EV-treated A549 and H460 cells. Moreover, H1299-EVs or H522-EVs led to greater number of cells arrested in the S phase and fewer cells arrested in the G1 phase (Fig. 1g), which explained, at least partially, why cancer cell-derived EVs promoted the cancer cell proliferation.

We further confirmed these findings in vivo using an established human NSCLC xenograft model. Seven days after the mice were injected with A549 cells, BEAS-2B-EVs or cancer cell-derived EVs (H1299-EVs or H522-EVs) were injected into the tumors, with the tumor growth recorded. The tumor growth curves showed that tumor growth of mice injected with untreated A549 cells were similar to that of mice injected with BEAS-2B-EV-treated A549 cells. The tumor growth was enhanced in mice treated with H1299-EVs or H522-EVs versus those in mice treated with BEAS-2B-EVs (Fig. 1h). The tumor volume and weight of mice treated with H1299-EVs or H522-EVs were higher than those in mice treated with BEAS-2B-EVs (Fig. 1i, j). In addition, we evaluated the proliferation of tumor cells by measuring the expression of proliferation marker, Ki-67, via IHC staining. The results showed that treatment with H1299-EVs or H522-EVs led to an increase in the positive expression of Ki-67 in cancer cells compared with treatment with BEAS-2B-EVs (Fig. 1k). Therefore, these findings suggested that cancer cell-derived EVs facilitated the proliferation of NSCLC cells in vitro and in vivo.

### Expression of miR-744 is decreased in cancer cell-derived EVs

**Fig. 2:**
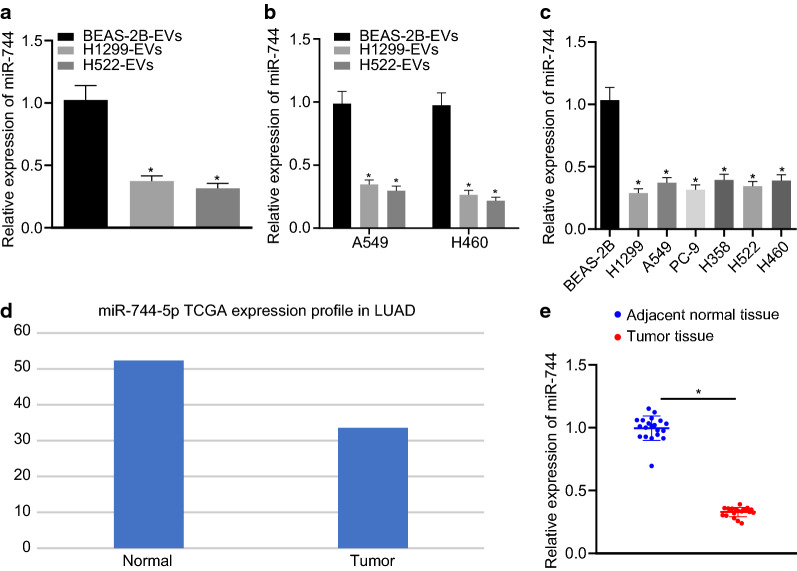
Cancer cell-derived EVs express low levels of miR-744. **a** Expression of miR-744 in EVs isolated from BEAS-2B, H1299 and H522 cells detected by RT-qPCR. **b** Expression of miR-744 determined by RT-qPCR in A549 and H460 cells treated with BEAS-2B-, H1299-, and H522-derived EVs (* *p* < 0.05 vs. A549 and H460 cells treated with BEAS-2B-derived EVs). **c** Expression of miR-744 determined by RT-qPCR in BEAS-2B and NSCLC cells (H1299, A549, PC-9, H358, H522, and H460) (* *p* < 0.05 vs. BEAS-2B cells). **d** Expression of miR-744 in tumor and normal samples in the TCGA database. The x-axis represents the sample type and the y-axis represents the mean expression value of miR-744. **e** Expression of miR-744 determined by RT-qPCR in clinical samples of tumor and adjacent normal tissues (* *p* < 0.05 vs. normal tissues). Data are summarized as mean ± standard deviation of three technical replicates. Data from two groups were compared using unpaired *t-*test and data from multiple groups were assessed by one-way ANOVA with Tukey's tests

According to the most recent studies, miR-744, which exerts an inhibitory effect on NSCLC proliferation and migration [13], is poorly expressed in cancer cell-derived EVs [14]. Here, we quantified the expression of miR-744 in BEAS-2B-EVs, H1299-EVs, and H522-EVs by RT-qPCR and found that the expression of miR-744 was lower in NSCLC cell (H1299 and H522)-derived EVs than that in normal bronchial epithelial cell (BEAS-2B)-derived EVs (Fig. 2a). These findings supported the notion that the different effects of normal cell-derived EVs and cancer cell-derived EVs on cancer cell biology are associated with the expression of miR-744. We then treated A549 and H460 cells with BEAS-2B-EVs, H1299-EVs, or H522-EVs and evaluated the expression of miR-744 in the cells. The result showed that as compared to treatment with BEAS-2B-EVs, treatment with H1299-EVs or H522-EVs led to lower levels of miR-744 in NSCLC cells (Fig. 2b). Furthermore, RT-qPCR revealed that miR-744 expression was reduced in NSCLC cell lines (H1299, A549, PC-9, H358, H522, and H460) when compared with BEAS-2B cells (Fig. 2c). The average expression value of miR-744-5p in lung cancer and normal samples included in TCGA was determined using a web-based database (http://bioinfo.life.hust.edu.cn/miR_path/download.html), and it was found that the expression of miR-744 was lower in lung cancer tissues than in normal tissues (Fig. 2d). For further verification, NSCLC clinical samples were collected and the miR-744 expression was quantified in 20 pairs of lung cancer tissues and normal adjacent tissues. The results verified that the expression of miR-744 was indeed decreased in tumor tissues (Fig. 2e). Collectively, these data indicated that miR-744 was expressed poorly in cancer cell-derived EVs.

### Overexpression of cancer cell-derived EV-delivered miR-744 inhibits NSCLC cell proliferation in vitro

**Fig. 3:**
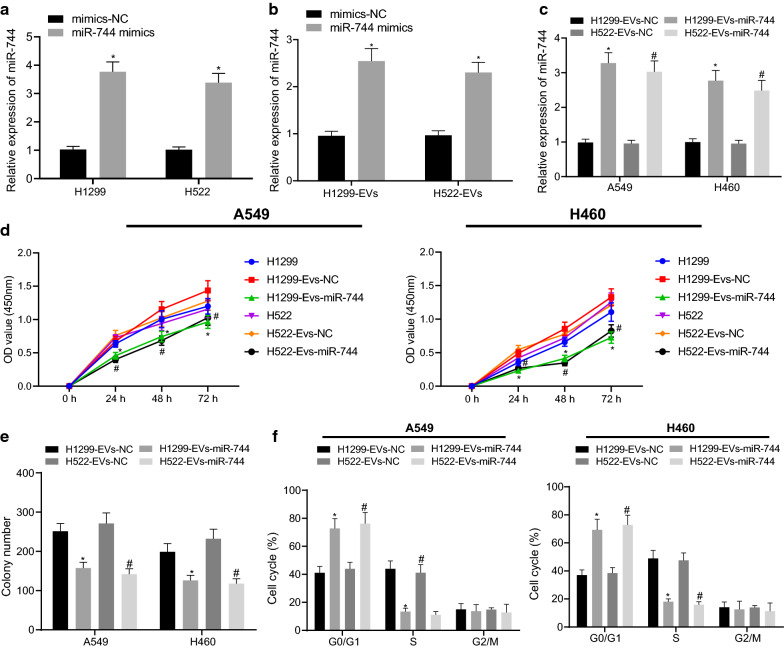
Highly expressed miR-744 in cancer cell-derived EVs represses NSCLC development in vitro*.* **a** Expression of miR-744 determined by RT-qPCR in H1299 and H522 cells transfected with miR-744 mimic (* *p* < 0.05 vs. H1299 or H522 cells transfected with mimic-NC). **b** Expression of miR-744 determined by RT-qPCR in EVs derived from H1299 or H522 cells treated with miR-744 mimic (* *p* < 0.05 vs. EVs derived from H1299 or H522 cells transfected with mimic-NC). **c** Expression of miR-744 determined by RT-qPCR in A549 and H460 cells treated with EVs derived from H1299 or H522 cells treated with miR-744 mimic. **d** CCK-8 proliferation assay evaluating the viability of A549 and H460 cells treated with EVs derived from untreated H1299 or H522 cells or H1299 or H522 cells transfected with miR-744 mimic. **e** Colony formation assay detecting the colony formation of A549 and H460 cells treated with EVs derived from H1299 or H522 cells transfected with miR-744 mimic. **f** Flow cytometry analysis showing the cell cycle distribution of A549 and H460 cells treated with EVs derived from H1299 or H522 cells transfected with miR-744 mimic (* *p* < 0.05, vs. A549 and H460 cells treated with EVs from H1299 cells transfected with mimic-NC; # *p* < 0.05, vs. A549 and H460 cells treated with EVs from H522 cells transfected with mimic-NC). Data are summarized as mean ± standard deviation of three technical replicates. Data from two groups were compared using unpaired *t-*test and data from multiple groups were assessed by one-way ANOVA with Tukey's post hoc tests. Data obtained at different time points were analyzed by Bonferroni-corrected repeated measures ANOVA

In order to confirm the hypothesis that aberrantly expressed miR-744 in cancer cell-derived EVs results in their different effects on NSCLC cell biology, the expression of miR-744 was modulated in NSCLC cells (H1299 and H522). We transfected H1299 and H522 cells with miR-744-mimic or mimic-NC and EVs were extracted from these transfcted cells (H1299-EVs-miR-744, H522-EVs-miR-744, H1299-EVs-NC, or H522-EVs-NC), after which the expression of miR-744 in these cells was measured by RT-qPCR. The resutls depicted that as compared to mimic-NC, miR-744-mimic increased the expression of miR-744 in both cells and EVs (Fig. 3a, b). A549 and H460 cells were treated with H1299-EVs-miR-744, H522-EVs-miR-744, H1299-EVs-NC, or H522-EVs-NC, which increased the expression of miR-744 in A549 and H460 cells (Fig. 3c). CCK-8 proliferation assay manifested that the viability of A549 and H460 cells treated with EVs from untreated H1299 and H522 cells did not differ from that of A549 and H460 cells treated with H1299-EVs-NC, or H522-EVs-NC, respectively (Fig. 3d). Besides, H1299-EVs-miR-744 and H522-EVs-miR-744 inhibited the viability (Fig. 3d) and colony formation (Fig. 3e) of A549 and H460 cells. Moreover, treatment of H1299-EVs-miR-744 or H522-EVs-miR-744 reduced the number of cells in the S phase and retained the cells in the G1 phase (Fig. 3f). In addition, we investigated whether EVs from BEAS-2B cells transfected with miR-744 inhibitor could have an effect on the phenotype of A549 and H460 cells. The results showed that BEAS-2B-EVs-miR-744 inhibitor could significantly increase the viability, migration, and invasion but decrease apoptosis of A549 and H460 cells (Additional file 1: Figure S1A-G). Overall, these findings indicated that overexpressed miR-744 delivered by cancer cell-derived EVs contributed to decreased cancer cell proliferaiton in vitro.

### Downregulation of miR-744 derived from cancer cell-derived EVs increased the expression of SUV39H1 in vitro

**Fig. 4:**
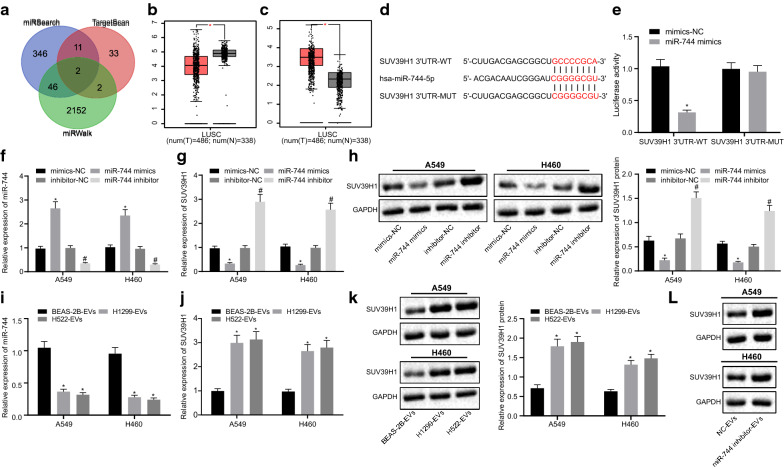
SUV39H1 is a direct target of cancer cell-derived EV miR-744. **a** Venn diagram showing the target genes of miR-744 predicted by three online tools (miRSearch, TargetScan, and miRWalk). (B and C) Data obtained from the TCGA database analyzed for the expression of BIN1 (**b**) and SUV39H1 (**c**) in lung tumor and normal samples. The x-axis represents the sample type and the y-axis represents the gene expression value. **d** Putative miR-744 binding sites in the 3′UTR of SUV39H1 mRNA in the TargetScan website (http://www.targetscan.org/vert_71/). **e** Dual-luciferase reporter assay validating the interaction between miR-744 and SUV39H1 in HEK293T cells. **f** and **g** Expression of miR-744 (**f**) and SUV39H1 (**g**) determined by RT-qPCR in A549 and H460 cells treated with miR-744 mimic or miR-744 inhibitor. **h** Western blot analysis of SUV39H1 protein in A549 and H460 cells transfected with miR-744 mimic or miR-744 inhibitor. **i** and **j** Expression of miR-744 (**i**) and SUV39H1 (**j**) determined by RT-qPCR in A549 and H460 cells treated with BEAS-2B-, H1299-, and H522-derived EVs. **k** Western blot analysis of SUV39H1 protein in A549 and H460 cells treated with BEAS-2B-, H1299-, and H522-derived EVs. **l** Western blot analysis of SUV39H1 protein in A549 and H460 cells treated with EVs isolated from the BEAS-2B cells transfected with miR-744 inhibitor. (* *p* < 0.05, vs. BEAS-2B-derived EVs, or A549, H460 cells, or HEK293T cells transfected with mimic-NC; # *p* < 0.05, vs. A549 and H460 cells transfected with inhibitor-NC). Data are shown as mean ± standard deviation of three technical replicates. Data from two groups were compared using unpaired *t-*test and data from multiple groups were compared by one-way ANOVA with Tukey's post hoc tests

Having demonstrated that upregulation of miR-744 in NSCLC cell-derived EVs repressed the proliferation of cancer cells, our focus shifted to investigate the underlying molecular mechanisms. Two genes, SUV39H1 and BIN1, were predicted as targets of miR-744 by three different online miRNA databases (miRSearch, TargetScan, and miRWalk) (Fig. 4a). We investigated their expression levels in lung cancer tissues using the GEPIA website which analyzes the data from the TCGA database (Fig. 4b, c). The results indicated that the expression of SUV39H1, a histone methyltransferase, was higher in lung cancer tissue than that in normal adjacent tissues, which opposed the expression trend of miR-744. In agreement, the expression of SUV39H1 has been shown to be upregulated in NSCLC tissues [15]. These findings highlighted the possibility of SUV39H1 as one of the targets of miR-744. To confirm this, miR-744 targeting sequence of SUV39H1 was predicted using the TargetScan website, which revealed the binding sites between miR-744 and the 3′UTR of SUV39H1 mRNA (Fig. 4d). Thereafter, a dual-luciferase reporter gene assay showed that the luciferase activity of SUV39H1-3′UTR-WT was decreased in HEK293T cells transfected with miR-744 mimic, while that of SUV39H1-3′UTR-MUT remained unaffected (Fig. 4e). These data indicated miR-744 might target the SUV39H1 gene. To further confirm this finding, we modulated the expression levels of miR-744 in NSCLC cells (A549 and H460) (Fig. 4f) and evaluated the expression of SUV39H1 at the transcriptional and translational levels in these cells (Fig. 4g, h). The results showed that miR-744 mimic resulted in decreased SUV39H1 mRNA (Fig. 4g) and protein expression (Fig. 4h), which was the opposite upon miR-744 inhibitor transfection. Importantly, we also noticed that the expression of miR-744 was lower in A549 and H460 cells treated with H1299-EVs or H522-EVs than that in cells with BEAS-2B-EV treatment (Fig. 4i). Consequently, the mRNA and protein expression levels of SUV39H1 were higher in A549 and H460 cells treated with H1299-EVs or H522-EVs than in cells following BEAS-2B-EV treatment (Fig. 4j, k). In addition, the isolated EVs from the BEAS-2B cells transfected with miR-744 inhibitor were used to incubate A549 and H460 cells, followed by the determination of SUV39H1 protein expression by western blot analysis. The results showed a significant increase of SUV39H1 protein expression in the A549 and H460 cells treated with EVs from miR-744 inhibitor-transfected BEAS-2B cells (Fig. 4l). These results suggested that miR-744 targeted SUV39H1, while downregulated miR-744 in cancer cell-derived EVs could lead to upregulated the expression of SUV39H1 in vitro in NSCLC cells.

### miR-744 encapsulated by cancer cell-derived EVs suppresses the development of NSCLC by targeting SUV39H1 in vitro

**Fig. 5:**
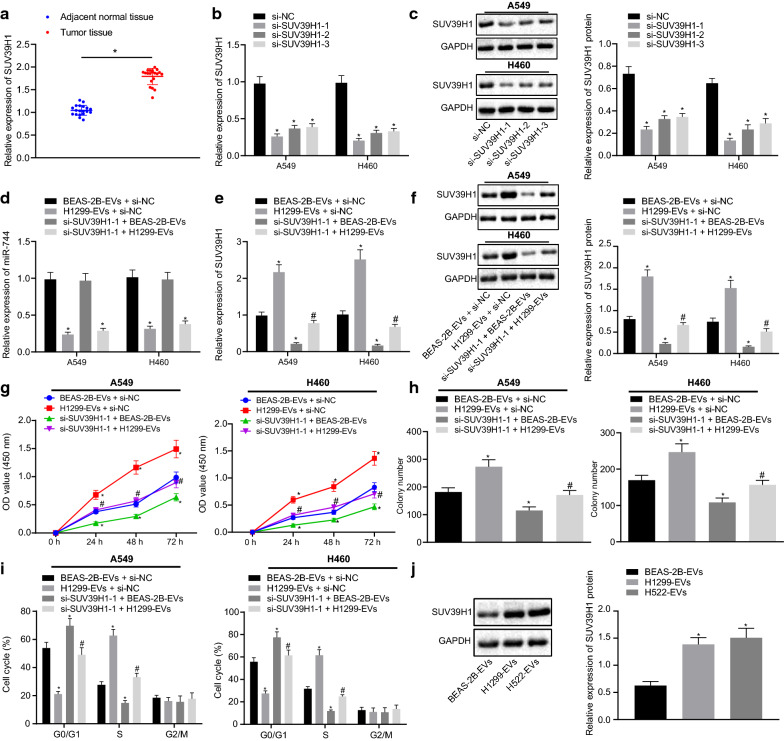
Cancer cell-derived EVs delivering miR-744 inhibits the development of NSCLC by targeting SUV39H1 in vitro*.* **a** Expression of SUV39H1 determined by RT-qPCR in cancer and adjacent normal tissues. (* *p* < 0.05, vs. adjacent normal tissues). **b** and **c** RT-qPCR (**b**) and western blot analysis (**c**) evaluating the knockdown efficiency of 3 siRNAs targeting SUV39H1 in A549 and H460 cells (* *p* < 0.05, vs. A549 and H460 cells transfected with si-NC). **d** Expression of miR-744 determined by RT-qPCR in si-SUV39H1-1-transfected A549 and H460 cells treated with BEAS-2B- and H1299-derived EVs. **e** and **f** RT-qPCR (**e**) and western blot analysis (**f**) measuring the expression of SUV39H1 in si-SUV39H1-1-transfected A549 and H460 cells treated with BEAS-2B- and H1299-derived EVs. **g**–**i** CCK-8 assay (**g**), colony formation assay (**h**), and cell cycle analysis (**i**) in si-SUV39H1-1-transfected A549 and H460 cells treated with BEAS-2B-, and H1299-derived EVs (* *p* < 0.05, vs. BEAS-2B-derived EVs, si-NC-transfected A549 and H460 cells treated with BEAS-2B-derived EVs; # *p* < 0.05, vs. si-NC-transfected A549 and H460 cells treated with H1299-derived EVs). **j** Western blots of SUV39H1 protein and its quantitation in tumor tissues of mice injected with A549 cells treated with BEAS-2B-, H522- and H1299-derived EVs (* *p* < 0.05, vs. mice injected with A549 cells treated with BEAS-2B-derived EVs). Data are summarized as mean ± standard deviation. Data from two groups were compared using unpaired *t-*test and data from multiple groups were compared by one-way ANOVA with Tukey's post hoc tests. Data obtained at different time points were compared by Bonferroni-corrected repeated measures ANOVA. Mice: n = 8/group. Each experiment was repeated three times

Our aforementioned findings suggested that EV-derived miR-744 targeted SUV39H1 and downregulated its expression in NSCLC cells. Thus, it was deemed possible that EV-derived miR-744 regulates the development of NSCLC by targeting SUV39H1. To validate this hypothesis, we first investigated the expression of SUV39H1 in clinical samples of lung tumor and normal adjacent tissues by RT-qPCR. The results showed markedly higher expression of SUV39H1 in lung tumor tissues than normal adjacent tissues (Fig. 5a). To explore the role of SUV39H1 in NSCLC development, we silenced SUV39H1 in NSCLC cells (A549 and H460) using siRNAs. The silencing efficiency was evaluated by RT-qPCR (Fig. 5b) and Western blot analysis (Fig. 5c), which revealed that siRNAs against SUV39H1 diminished SUV39H1 expression in A549 and H460 cells. Here, si-SUV39H1-1 exhibited superior silencing efficiency, and, was therefore, used in further experiments. Then A549 and H460 cells were treated with EVs from BEAS-2B or H1299 cells and then transfected with si-SUV39H1-1. A lower level of miR-744 was noted in A549 and H460 cells treated with H1299-EVs + si-SUV39H1-1 compared to cells treated with BEAS-2B-EVs + si-SUV39H1-1 (Fig. 5d). Moreover, compared to treatment with BEAS-2B-EVs + si-NC, SUV39H1 expression was elevated in A549 and H460 cells treated with H1299-EVs + si-NC, which was negated by treatment with H1299-EVs + si-SUV39H1-1. In contrast to treatment with H1299-EVs + si-NC, treatment with si-SUV39H1-1 + BEAS-2B-EVs resulted in the decline of SUV39H1 expression in A549 and H460 cells (Fig. 5e, f). Next, we investigated the effect of SUV39H1 silencing on EV-derived miR-744 mediated NSCLC cell proliferation using CCK-8 assay and colony formation assay. The results showed that the proliferation and colony formation in BEAS-2B-EV-treated A549 and H460 cells were retarded by si-SUV39H1-1 treatment. At the same time, A549 and H460 cells displayed enhanced proliferation and colony formation following H1299-EVs + si-NC treatment, when compared with that in BEAS-2B-EVs + si-NC treatment. However, this promotive effect on cell proliferation and colony formation was countered by further si-SUV39H1-1 treatment (Fig. 5g, h). Cell cycle analysis revealed that si-SUV39H1-1 reduced the A549 and H460 cells arrested in the S phase and increased the number of cells arrested in the G1 phase in the presence of BEAS-2B-EVs. Moreover, upon treatment with H1299-EVs + si-NC, greater numbers of A549 and H460 cells were retained in the S phase while cells in the G1 phase were reduced, when compared to treatment with BEAS-2B-EVs + si-NC. However, additional treatment of si-SUV39H1-1 diminished this effect (Fig. 5i). We also investigated SUV39H1 expression levels in A549 xenografts and found that upon treatment with H1299-EVs or H522-EVs, the expression of SUV39H1 was enhanced in tumor tissues of mice as compared to that upon treatment with BEAS-2B-EVs (Fig. 5j). In order to exclude the out-off target effect of the selected siRNA, A549 and H460 cells were treated with EVs from BEAS-2B or H1299 cells and then transfected with si-SUV39H1-2 to observe functional changes in A549 and H460 cells. The results were consistent with those obtained following si-SUV39H1-1 transfection (Additional file 2: Figure S2A-F). Altogether, these findings suggested that cancer cell-derived EV-encapsulated miR-744 reduced the development of NSCLC by targeting SUV39H1.

### SUV39H1 regulates the expression of Smad9/BMP4 in vitro

**Fig. 6:**
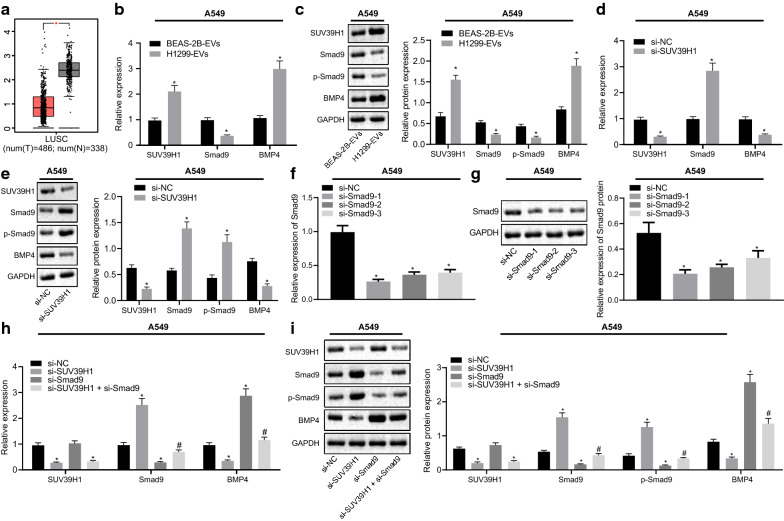
SUV39H1 regulates the expression of Smad9/BMP4 in A549 cells. **a** Data obtained from the TCGA database analyzed for the expression levels of Smad9 in lung tumor and normal samples. The x-axis represents the sample type and the y-axis represents the gene expression value. **b** Expression levels of SUV39H1, Smad9, and BMP4 determined by RT-qPCR in A549 cells treated with EVs from BEAS-2B and H1299 cells. **c** Western blot analysis showing the expression of SUV39H1, Smad9, BMP4 and the extent of Smad9 phosphorylation in A549 cells treated with EVs from BEAS-2B and H1299 cells (* *p* < 0.05, vs. A549 cells treated with EVs from BEAS-2B cells). **d** Expression of SUV39H1, Smad9, and BMP4 determined by RT-qPCR in si-SUV39H1-treated A549 cells. **e** Western blot analysis evaluating the expression of SUV39H1, Smad9, and BMP4 and the extent of Smad9 phosphorylation in si-SUV39H1-treated A549 cells (* *p* < 0.05, vs. A549 cells treated with si-NC). **f** Knockdown efficiency of siRNA targeting Smad9 determined by RT-qPCR in A549 cells. **g** Western blot analysis validating the knockdown efficiency of siRNA targeting Smad9 in A549 cells (* *p* < 0.05, vs. A549 cells treated with si-NC). **h** Expression of SUV39H1, Smad9, and BMP4 in A549 cells transfected with si-Smad9, si-SUV39H1 or both. **i** Western blot analysis detecting the expression of SUV39H1, Smad9, and BMP4 and the extent of Smad9 phosphorylation in A549 cells transfected with si-Smad9, si-SUV39H1 or both (* *p* < 0.05, vs. A549 cells treated with si-NC; # *p* < 0.05, vs. A549 cells treated with si-Smad9). Data are summarized as mean ± standard deviation of three technical replicates. Data from two groups were compared using unpaired *t-*test and data from multiple groups were analyzed by one-way ANOVA with Tukey's post hoc tests

SUV39H1 has been reported to inhibit the expression of Smad9 [16], which has been shown to suppress the expression of BMP4 [17]. Moreover, BMP4 has been documented to promote the initiation of NSCLC [18] and is highly expressed in NSCLC tumor tissues [24]. Thus, we speculated that reduced expression of miR-744 in cancer cell-derived EVs enhanced SUV39H1, which further suppressed the expression of Smad9 and consequently elevated the BMP4 expression, promoting the NSCLC progression. By analyzing the expression of Smad9 in NSCLC tumor tissues using data obtained from the TCGA database, we found that Smad9 was poorly expressed in NSCLC tumor tissue samples (Fig. 6a). We then evaluated the expression of SUV39H1, Smad9, and BMP4 inA549 and H460 cells treated with BEAS-2B-EVs or H1299-EVs by RT-qPCR and western blot analysis. The findings showed that cells treated with H1299-EVs expressed higher levels of SUV39H1 and BMP4 but lower Smad9 levels and extent of Smad9 phosphorylation (Fig. 6b, c, and Additional file 3: Figure S3A, B). We also observed that silencing SUV39H1 decreased the expression of BMP4 but increased Smad9 expression and the extent of Smad9 phosphorylation in A549 and H460 cells (Fig. 6d, e and Additional file 3: Figure S3C, D). This indicated that reduction of miR-744 resulted in upregulated SUV39H1 expression, which, in turn, could upregulate BMP4 expression by suppressing the expression of Smad9 in NSCLC cells. In order to validate our hypothesis that SUV39H1 regulates the expression of BMP4 in NSCLC cells by suppressing Smad9 expression, Smad9 was silenced in A549 and H460 cells and the knockdown efficiency was evaluated by RT-qPCR (Fig. 6f, Additional file 3: Fig. 3e) and western blot analysis (Fig. 6g, Additional file 3: Figure S3F). The results manifested that siRNAs against Smad9 diminished Smad9 expression in A549 and H460 cells, and that si-Smad9-1 exhibited the highest silencing efficiency and was therefore used in further experiments. We then investigated the expression of BMP4 in A549 and H460 cells in which SUV39H1 and/or Smad9 silencing was performed. We found that, SUV39H1 knockdown (si-SUV39H1) resulted in upregulated Smad9 expression and extent of Smad9 phosphorylation, while it downregulated the expression of SUV39H1 and BMP4 in A549 and H460 cells. However, silencing of Smad9 led to the opposite effects except for unchanged SUV39H1 expression in A549 and H460 cells. Moreover, concomitant silencing of SUV39H1 and Smad9 (si-SUV39H1 + si-Smad9) reduced the expression levels of Smad9 and extent of Smad9 phosphorylation but increased BMP4 expression in A549 and H460 cells in contrast to treatment with si-SUV39H1 (Fig. 6h, i and Additional file 3: Fig. S3G, H). These results suggested that the regulatory role of SUV39H1 on BMP4 depended upon Smad9 in vitro.

### Downregulated miR-744 in cancer cell-derived EVs promotes the development of NSCLC by regulating the SUV39H1/Smad9/BMP4 axis

**Fig. 7:**
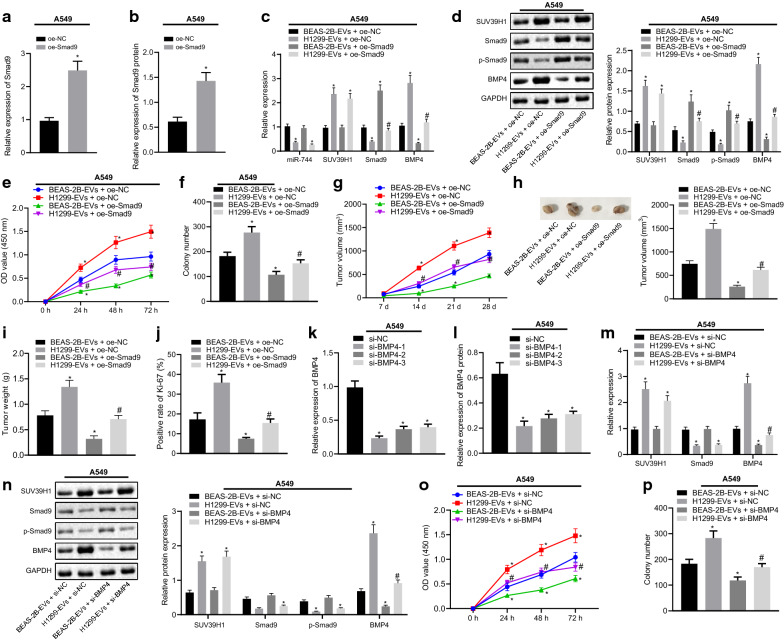
miR-744 transferred by cancer cell-derived EVs represses the development of NSCLC by regulating the SUV39H1/Smad9/BMP4 axis in A549 cells. **a** Overexpression efficiency of Smad9 determined by RT-qPCR in A549 cells. **b** Western blot analysis validating the overexpression efficiency of Smad9 in A549 cells (* *p* < 0.05, vs. OE-NC-treated A549 cells). **c** Expression of miR-744, SUV39H1, Smad9, and BMP4 determined by RT-qPCR in OE-Smad9-transfected A549 cells treated with EVs from BEAS-2B and H1299 cells. **d** Representative Western blots of SUV39H1, Smad9, and BMP4 and p-Smad9 and their quantitation in OE-Smad9-transfected A549 cells treated with EVs from BEAS-2B and H1299 cells. **e** Viability of OE-Smad9-transfected A549 cells treated with EVs from BEAS-2B and H1299 cells measured using CCK-8 assay. **f** Colony formation of OE-Smad9-transfected A549 cells treated with EVs from BEAS-2B and H1299 cells measured using colony formation assay (* *p* < 0.05, vs. si-NC-transfected A549 cells treated with EVs from BEAS-2B cells; # *p* < 0.05, vs. si-NC-transfected A549 cells treated with EVs from H1299 cells). **g**–**j** Results of in vivo tumor growth*.* A549 cells with or without Smad9 overexpression were injected into mice to establish the xenograft model and the EVs were injected into the tumors 7 days after transplantation. Tumor volume (**g**) was measured every 3 days; and final tumor volume (**h**) and weight (**i**) were measured, averaged, and compared after 4 weeks. The expression of proliferation marker, Ki-67 (**j**), was measured by IHC staining images were taken (* *p* < 0.05, vs. mice injected with A549 cells treated with BEAS-2B-EVs; # *p* < 0.05, vs. mice injected with A549 cells treated with H1299-EVs). **k** Knockdown efficiencies of 3 siRNAs targeting BMP4 determined by RT-qPCR in A549 cells. **l** Western blot analysis validating the knockdown efficiencies of 3 siRNAs targeting BMP4 in A549 cells (* *p* < 0.05, vs. A549 cells transfected with si-NC). **m** Expression of SUV39H1, Smad9, and BMP4 in si-BMP4-transfected A549 cells treated with EVs from BEAS-2B and H1299 cells. **n** Western blots of SUV39H1, Smad9, BMP4 and p-Smad9 and their quantitation in si-BMP4-transfected A549 cells treated with EVs from BEAS-2B and H1299 cells. **o** Viability of si-BMP4-transfected A549 cells treated with EVs from BEAS-2B and H1299 cells measured using CCK-8 assay. **p** Colony formation of si-BMP4-transfected A549 cells treated with EVs from BEAS-2B and H1299 cells measured using colony formation assay (* *p* < 0.05, vs. si-NC-transfected A549 cells treated with EVs from BEAS-2B cells; # *p* < 0.05, vs. si-NC-transfected A549 cells treated with EVs from H1299 cells). Data are summarized as mean ± standard deviation. Data from two groups were compared using unpaired *t-*test and data from multiple groups were assessed by one-way ANOVA with Tukey's tests. Data obtained at different time points were assessed by Bonferroni-corrected repeated measures ANOVA. Mice: n = 8/group. Each experiment was repeated three times

In order to define the role of Smad9/BMP4 in NSCLC development, we overexpressed Smad9 (OE-Smad9) in A549 and H460 cells. The overexpression was validated by RT-qPCR (Fig. 7a, Additional file 4: Figure S4A) and western blot analysis (Fig. 7b, Additional file 4: Figure S4B). We then treated OE-Smad9-transfected A549 and H460 cells with BEAS-2B- or H1299-derived EVs, and examined the expression of miR-744, SUV39H1, Smad9, and BMP4 by RT-qPCR (Fig. 7c, Additional file 4: Figure S4C) or western blot analysis (Fig. 7d, Additional file 4: Figure S4D). In the presence of BEAS-2B-EVs, OE-Smad9 treatment increased Smad9 and extent of Smad9 phosphorylation but decreased BMP4 expression in A549 and H460 cells. H1299-EVs + OE-NC-treated A549 and H460 cells displayed higher expression of SUV39H1 and BMP4, and lower expression of Smad9 and extent of Smad9 phosphorylation, compared to BEAS-2B-EVs + OE-NC-treated A549 and H460 cells. Interestingly, H1299-EVs-mediated upregulation of BMP4 could be diminished upon overexpression of Smad9. Thus, cancer cell-derived EVs could upregulate BMP4 expression by suppressing Smad9 expression in NSCLC cells. We then addressed whether cancer cell-derived EVs promoted NSCLC cell growth by regulating the Smad9/BMP4 pathway and thus, CCK-8 proliferation assay (Fig. 7e, Additional file 4: Figure S4E) and colony formation assay (Fig. 7f, Additional file 4: Figure S4F) were performed. The results showed a reduction of viability and colony formation of A549 and H460 cells in response to Smad9 overexpression in the presence of BEAS-2B-EVs. Higher viability and colony formation were observed in H1299-EVs + OE-NC-treated A549 and H460 cells than in BEAS-2B-EVs + OE-NC-treated cells. However, the promotive effects of H1299-EVs on cell proliferation and colony formation were compromised by Smad9 overexpression. Similar results were observed in the mouse xenograft model. The tumor growth was observed to be faster in mice injected with A549 cells treated with H1299-derived EVs than in mice injected with A549 cells treated with BEAS-2B-derived EVs. However, tumor growth promoted by H1299-EVs was inhibited by Smad9, while in the presence of BEAS-2B-EVs, overexpression of Smad9 reduced tumor growth in mice (Fig. 7g). Moreover, tumor weight and volume were higher in H1299-EV-treated mice but lower in mice treated with BEAS-2B-EVs + Smad9 than in BEAS-2B-EV-treated mice. However, H1299-EVs failed to increase the tumor weight and volume in the tumors after Smad9 was stably overexpressed (Fig. 7h, i). Furthermore, we also evaluated the expression of proliferation marker, Ki-67, in the tumor tissues by IHC analysis, which showed that H1299-EV treatment led to an upregulation of Ki-67 positive expression but treatment with BEAS-2B-EVs + Smad9 diminished Ki-67 positive expression when compared with BEAS-2B-EV treatment (Fig. 7j). On the other hand, H1299-EVs failed to elevate the expression of Ki-67 in the tumors once Smad9 was stably overexpressed. These findings indicated that cancer cell-derived EVs promoted NSCLC development in a Smad9 dependent manner.

Thereafter, we silenced BMP4 by using siRNAs and the knockdown efficiency was evaluated by RT-qPCR (Fig. 7k, Additional file 4: Figure S4G) and western blot analysis (Fig. 7l, Additional file 4: Figure S4H). siRNAs against BMP4 could diminish BMP4 expression, and si-BMP4-1 achieved the highest knockdown efficiency and was thus selected for further experiments. A549 and H460 cells were treated with si-BMP4 and BEAS-2B-EVs or H1299-EVs and the expression of SUV39H1, Smad9, and BMP4 was evaluated by RT-qPCR (Fig. 7m, Additional file 4: Figure S4I) and western blot analysis (Fig. 7n, Additional file 4: Figure S4J). The results showed that higher expression of SUV39H1 and BMP4, and lower expression of Smad9 and extent of Smad9 phosphorylation in H1299-EVs + si-NC-treated A549 and H460 cells compared with that in BEAS-2B-EVs + si-NC-treated A549 and H460 cells. In the presence of BEAS-2B-EVs, si-BMP4 contributed to decline of BMP4 expression in A549 and H460 cells. We also observed that siRNA targeting BMP4 could decrease the expression of H1299-EV-upregulated BMP4. Functional assays showed that compared with BEAS-2B-EVs + si-NC-treated A549 and H460 cells, viability (Fig. 7o, Additional file 4: Figure S4K) and colony formation (Fig. 7p, Additional file 4: Figure S4L) were enhanced in H1299-EVs + si-NC-treated cells, but the trends were opposite in A549 and H460 cells after treatment with BEAS-2B-EVs + si-BMP4. However, when BMP4 was silenced, H1299-EV treatment was not able to promote the viability and colony formation of A549 and H460 cells. These findings suggested that reduced expression of miR-744 by cancer cell-derived EVs enhanced the expression of SUV39H1, which further regulated Smad9/BMP4 to promote NSCLC development.

## Discussion

As a leading cause of cancer-related mortality in the world, lung cancer is the most frequently diagnosed malignancy [25]. Based on the histological features, lung cancer can be classified into two types: small-cell lung cancer (SCLC) and NSCLC. NSCLC accounts for around 85% of total lung cancer cases [26]. The standard approach for NSCLC treatment is curative surgical resection in combination with or without chemotherapy or chemoradiotherapy for patients at an early stage [27]. However, most patients with NSCLC are diagnosed at the late stage which leads to poor prognosis [28]. Therefore, more effective therapeutic strategies should be developed which necessitate a better understanding of the molecular pathogenesis of this disease. In this study, we focused on the role of cancer cell-derived EVs in tumorigenesis and aimed at identifying novel potential therapeutic targets.

We first observed a decreased expression of miR-744 in tumor cell-derived EVs compared with normal lung epithelial cell-derived EVs. This finding is in consistent with a previous study demonstrating that tumor-derived EVs presented with downregulated miR-744 expression [29]. Thus, miR-744 could be a potential major factor that regulates tumor-derived EV-mediated tumorigenesis. The present study also found that reduced miR-744 expression in cancer cell-derived EVs stimulated NSCLC cell proliferaiton. However, results reported by Sha et al. [30] are contrary to ours. This may be due to two specific reasons: firstly, Sha et al. did not determine whether miR-744 expression changes could affect the proliferation or invasion of NSCLC cells and secondly, they have focused on the interaction between Jun and miR-744 in its effect on NSCLC cells. Once Jun was over-activated, miR-744 expression would be aberrant, which might increase the possibility of NSCLC proliferation. Taking these findings together with ours, it is evident that the low expression or abnormally high expression of miR-744 leads to opposite effects on NSCLC cells. Recent studies have found that miR-744 has a strong anti-cancer effect in thyroid cancer and liver cancer [31, 32], which is consistent with our findings. Therefore, the effect of miR-744 on the function of NSCLC cells is worthy of further exploration.

A previous study has demonstrated that upregulated SUV39H1 facilitates cancer cell proliferation, enhances migratory potency of cancer cells, and suppresses cancer cell apoptosis [15]. In our study, we uncovered upregulated SUV39H1 expression in NSCLC tissues and cells, and showed that SUV39H1 was a direct target of miR-744. More importantly, we observed that normal lung epithelial cell-derived EVs treated cells express lower levels of SUV39H1 compared with cells treated by NSCLC cell-derived EVs. Our data also demonstrated that miR-744 shuttled by NSCLC cell-derived EVs arrested the proliferation of cancer cells by inhibiting SUV39H1 expression. Similarly, emerging evidence demonstrates that miRNAs play an important role in regulating cancer cell growth, invasion and metastasis by inhibiting the expression of their targets [33]. Interestingly, accruing evidence has shown that SUV39H1 negatively regulates the expression and activity of BMP4, which is closely related to NSCLC development and progression [18]. This evidence indicated the possibility that in NSCLC, upregulated SUV39H1 may contribute to tumor progression by regulating BMP4. In this study, we noticed that SUV39H1 silencing downregulated the expression of BMP4, which validated the aforementioned assumption. BMP4 expression has been shown to be negatively regulated by Smad9 [17]. Further evidence has suggested that SUV39H1 exerts a suppressive effect on Smad9 expression [16]. Moreover, the expression of BMP4 is higher in NSCLC tumors than that in paired adjacent non-tumor tissues [24]. In alignment with these studies, our data showed that when SUV39H1 was silenced in NSCLC cells, the expression of Smad9 was upregulated and the expression of BMP4 was downregulated. Consistently, NSCLC cell-derived EVs resulted in increased SUV39H1 and BMP4 expression levels, and decreased Smad9 expression in their target cells compared with those stimulated by normal lung epithelial cell-derived EVs.

**Fig. 8:**
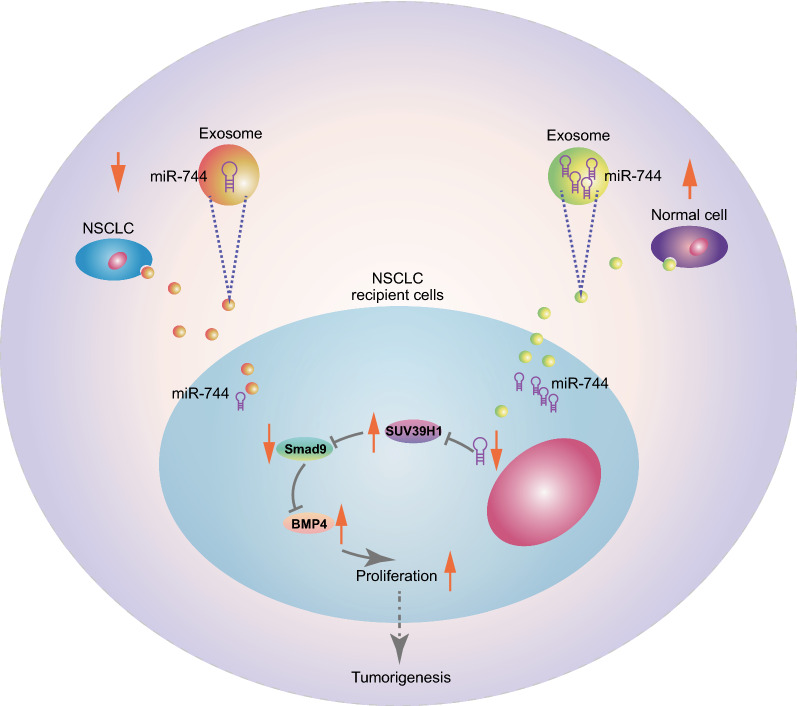
Schematic diagram showing NSCLC cell-derived EVs encompassing decreased miR-744 expression enhance the expression of SUV39H1, which suppresses the expression of Smad9, and enhances the BMP4 activity, consequently promoting NSCLC development

Taken together, our data suggested that downregulated miR-744 in NSCLC cell-derived EVs enhanced the expression of SUV39H1, which then suppressed BMP4 expression by inhibiting Smad9, in NSCLC cells. Functionally, we demonstrated that, in vivo and in vitro, tumor cell-derived EVs facilitated NSCLC tumor growth as reflected by increased viability, enhanced colony formation potency, and higher cell population in S phase (Fig. 8).

## Conclusion

In conclusion, our study has uncovered a previous uncharacterized mechanism by which reduction of miR-744 in tumor-derived EVs promoted NSCLC development. These findings may be of significant great translational value towards improving NSCLC diagnosis and treatment. Firstly, we verified that miR-744 is downregulated in tumor-derived EVs, as has been previously reported [13]. Therefore, our data support the suggestion that miR-744 could be used as a biomarker for NSCLC early diagnosis [14]. Secondly, in this study we have identified a previous unreported mechanism by which miR-744 from tumor derived EV contributes to the pathogenesis of NSCLC. This finding will help us to better understand the pathophysiology of NSCLC and develop more effective therapeutic approaches.

## Supplementary Information

**Additional file 1: Figure S1**. Poorly expressed miR-744 in cancer cells-derived EVs promotes NSCLC development. (A). Expression of miR-744 determined by RT-qPCR in BEAS-2B cells transfected with miR-744 inhibitor (* *p* < 0.05 vs. BEAS-2B cells transfected with inhibitor-NC). (B) Expression of miR-744 determined by RT-qPCR in EVs isolated from the BEAS-2B cells transfected with miR-744 inhibitor (* *p* < 0.05 vs. EVs isolated from the BEAS-2B cells transfected with inhibitor-NC). (C) Expression of miR-744 determined by RT-qPCR in A549 and H460 cells treated with EVs isolated from the BEAS-2B cells transfected with miR-744 inhibitor. (D). Viability of A549 and H460 cells treated with EVs isolated from the BEAS-2B cells transfected with miR-744 inhibitor measured by CCK-8 assay. (E) Apoptosis of A549 and H460 cells treated with EVs isolated from the BEAS-2B cells transfected with miR-744 inhibitor measured by flow cytometry. (F) Invasion of A549 and H460 cells treated with EVs isolated from the BEAS-2B cells transfected with miR-744 inhibitor measured by scratch test. (G) Migration of A549 and H460 cells treated with EVs isolated from the BEAS-2B cells transfected with miR-744 inhibitor measured by Transwell assay (* *p* < 0.05, vs. A549 and H460 cells treated with EVs isolated from the BEAS-2B cells transfected with NC). Data are summarized as mean ± standard deviation of three technical replicates. Data from two groups were compared using unpaired *t-*test and data obtained at different time points was compared by Bonferroni-corrected repeated measures ANOVA.

**Additional file 2: Figure S2.** Cancer cell-derived EV-encapsulated miR-744 affects NSCLC development by targeting SUV39H1. (A). Expression of miR-744 determined by RT-qPCR in A549 and H460 cells treated with EVs from BEAS-2B or H1299 cells and si-SUV39H1-2. (B) Expression of SUV39H1 determined by RT-qPCR in A549 and H460 cells treated with EVs from BEAS-2B or H1299 cells and si-SUV39H1-2. (C), Representative Western blots of SUV39H1 protein and its quantitation in A549 and H460 cells treated with EVs from BEAS-2B or H1299 cells and si-SUV39H1-2. (D). Viability of A549 and H460 cells treated with EVs isolated from BEAS-2B or H1299 cells and si-SUV39H1-2 measured by CCK-8 assay. (E) Colony formation of A549 and H460 cells treated with EVs from BEAS-2B or H1299 cells and si-SUV39H1-2 measured by colony formation assay. (F) Cell cycle distribution of A549 and H460 cells treated with EVs from BEAS-2B or H1299 cells and si-SUV39H1-2 measured by flow cytometry. * *p* < 0.05, vs. A549 and H460 cells treated with EVs isolated from BEAS-2B or H1299 cells and si-NC. # *p* < 0.05, vs. A549 and H460 cells treated with EVs isolated from H1299 cells and si-NC. Data are summarized as mean ± standard deviation of three technical replicates. Data comparisons between two groups were made using unpaired *t-*test and data from multiple groups were compared by one-way ANOVA with Tukey's post hoc tests. Data from multiple groups was compared by Bonferroni-corrected repeated measures ANOVA.

**Additional file 3: Figure S3.** SUV39H1 regulates the expression of Smad9/BMP4 in H460 cells. (A) Expression of SUV39H1, Smad9, and BMP4 determined by RT-qPCR in H460 cells treated with EVs from BEAS-2B and H1299 cells. (B) Western blot analysis showing the expression of SUV39H1, Smad9, BMP4 and the extent of Smad9 phosphorylation in H460 cells treated with EVs from BEAS-2B and H1299 cells (* *p* < 0.05, vs. H460 cells treated with EVs from BEAS-2B cells). (C) Expression of SUV39H1, Smad9, and BMP4 determined by RT-qPCR in si-SUV39H1-treated H460 cells. (D) Western blot analysis evaluating the expression levels of SUV39H1, Smad9, and BMP4 and the extent of Smad9 phosphorylation in si-SUV39H1-treated H460 cells (* *p* < 0.05, vs. H460 cells treated with si-NC). (E) Knockdown efficiency of 3 siRNAs targeting Smad9 determined by RT-qPCR in H460 cells. (F) Western blot analysis validating the knockdown efficiency of 3 siRNAs targeting Smad9 in H460 cells (* *p* < 0.05, vs. H460 cells treated with si-NC). (G) Expression levels of SUV39H1, Smad9, and BMP4 in H460 cells transfected with si-Smad9, si-SUV39H1 or both. (H) Western blot analysis detecting the expression levels of SUV39H1, Smad9, and BMP4 and the extent of Smad9 phosphorylation in H460 cells transfected with si-Smad9, si-SUV39H1 or both. (* *p* < 0.05, vs. H460 cells treated with si-NC; # *p* < 0.05, vs. H460 cells treated with si-Smad9). Data are summarized as mean ± standard deviation of three technical replicates. Data comparisons between two groups were made using unpaired *t-*test and data from multiple groups were compared by one-way ANOVA with Tukey's tests.

**Additional file 4: Figure S4.** Downregulated miR-744 transferred by cancer cell-derived EVs promotes the development of NSCLC by regulating the SUV39H1/Smad9/BMP4 axis in H460 cells. (A) Overexpression efficiency of Smad9 determined by RT-qPCR in H460 cells. (B) Western blot analysis validating the overexpression efficiency of Smad9 in H460 cells (* *p* < 0.05, vs. OE-NC-treated H460 cells). (C). Expression of miR-744, SUV39H1, Smad9, and BMP4 determined by RT-qPCR in OE-Smad9-transfected H460 cells treated with EVs from BEAS-2B and H1299 cells. (D) Representative Western blots of SUV39H1, Smad9, and BMP4 and p-Smad9 and their quantitation in OE-Smad9-transfected H460 cells treated with EVs from BEAS-2B and H1299 cells. (E) Viability of OE-Smad9-transfected H460 cells treated with EVs from BEAS-2B and H1299 cells measured using CCK-8 assay. (F) Colony formation of OE-Smad9-transfected H460 cells treated with EVs from BEAS-2B and H1299 cells measured using colony formation assay (* *p* < 0.05, vs. si-NC-transfected H460 cells treated with EVs from BEAS-2B cells; # *p* < 0.05, vs. si-NC-transfected H460 cells treated with EVs from H1299 cells). (G) Knockdown efficiencies of 3 siRNAs targeting BMP4 determined by RT-qPCR in H460 cells. (H) Western blot analysis validating the knockdown efficiencies of 3 siRNAs targeting BMP4 in H460 cells (* *p* < 0.05, vs. H460 cells transfected with si-NC). (I) Expression of SUV39H1, Smad9, and BMP4 in si-BMP4-transfected H460 cells treated with EVs from BEAS-2B and H1299 cells. (J) Representative Western blots of SUV39H1, Smad9, BMP4 and p-Smad9 and their quantitation in si-BMP4-transfected H460 cells treated with EVs from BEAS-2B and H1299 cells. (K) Viability of si-BMP4-transfected H460 cells treated with EVs from BEAS-2B and H1299 cells measured using CCK-8 assay. (L) Colony formation of si-BMP4-transfected H460 cells treated with EVs from BEAS-2B and H1299 cells measured using colony formation assay. (* *p* < 0.05, vs. si-NC-transfected H460 cells treated with EVs from BEAS-2B cells; # *p* < 0.05, vs. si-NC-transfected H460 cells treated with EVs from H1299 cells). Data are summarized as mean ± standard deviation of three technical replicates. Data comparisons between two groups were made using unpaired *t-*test and data from multiple groups were compared using one-way ANOVA with Tukey's post hoc tests. Data from multiple time-point groups were compared by Bonferroni-corrected repeated measures ANOVA.

## Data Availability

The primary data for this study is available from the authors on direct request.

## Abbreviations

NSCLC: Non-small cell lung cancer
EVs: Extracellular vehicles
miRNAs: MicroRNAs
LMO7: LIM-domain-only protein 7
BMP4: Bone morphogenetic protein 4
SUV39H1: Suppressor of variegation 3-9 homolog 1
NTA: Nanopartical tracking analysis
TEM: Transmission electron microscopy
PI: Propidium iodide
FITC: Fluorescein isothiocyanate
FBS: Fetal bovine serum
OE: Overexpression
CCK-8: Cell counting kit-8 assay
OD: Optical density
NC: Negative control
Smad9: SMAD family member 9
siRNA: Small interfering RNA
3′UTR: 3′Untranslated region
PBS: Phosphate-buffered saline
RT-qPCR: Reverse transcription quantitative polymerase chain reaction
GAPDH: Glyceraldehyde-3-phosphate dehydrogenase
p: Phosphorylated
HE: Hematoxylin and eosin
IHC: Immunohistochemistry
WT: Wild-type
mut: Mutant
IgG: Immunoglobulin G
ANOVA: Analysis of variance
SCLC: Small-cell lung cancer
